# Immune checkpoint inhibitors and the inflammatory dysregulation: decoding cutaneous and gastrointestinal immune-related adverse events for the allergist and immunologist

**DOI:** 10.3389/fimmu.2026.1914702

**Published:** 2026-09-29

**Authors:** Muhammad Wasim, Guoda Ma, Yajun Wang

**Affiliations:** 1 Maternal and Children’s Health Research Institute, Shunde Women and Children’s Hospital, Guangdong Medical University, Foshan, China; 2 Department of Pediatrics, Shunde Women and Children Hospital, Guangdong Medical University, Foshan, China

**Keywords:** anti-CTLA-4, anti-PD-1, atopy, immune checkpoint inhibitors (ICIs), immune-related adverse events (irAEs)

## Abstract

Immune checkpoint inhibitors (ICIs) targeting programmed cell death protein 1 (PD-1), its ligand (PD-L1), and cytotoxic T-lymphocyte-associated protein 4 (CTLA-4) have revolutionized oncology, yet they unleash a unique spectrum of inflammatory toxicities known as immune-related adverse events (irAEs). Cutaneous and gastrointestinal irAEs represent the most frequent and clinically challenging manifestations encountered by allergists and clinical immunologists. Unlike classical type I–IV hypersensitivity reactions, irAEs arise from disruption of peripheral immune tolerance, leading to T-cell hyperactivation, B-cell dysregulation with IgE and IgG4 skewing, and pro-inflammatory cytokine networks dominated by interleukin (IL)-6, IL-17, and interferon-gamma. IrAEs are best conceptualized as a distinct category of drug-induced autoimmunity, rather than a fifth type of hypersensitivity reaction. This review provides a comprehensive, clinically actionable framework for the practicing allergist. We dissect the divergent pathophysiology of anti-CTLA-4 versus anti-PD-1 toxicities, characterize the full spectrum of cutaneous eruptions and delineate the grading and management of ICI-induced colitis, which mimics inflammatory bowel disease but has distinct immune signatures. We critically evaluate emerging biomarkers (eosinophilia, serum IL-6, and fecal calprotectin) and present an evidence-based algorithm for step-up therapy: from topical corticosteroids and antihistamines for grade 1–2 rash, to systemic corticosteroids for grade 2–3 colitis, and biologic agents (infliximab, vedolizumab, tocilizumab, dupilumab) for corticosteroid-refractory cases. A dedicated section tackles the allergist’s most challenging dilemma (rechallenge after severe irAEs) providing evidence-based guidance. We identify critical knowledge gaps: pre-existing atopy as a risk factor, antihistamine prophylaxis utility, and overlapping toxicity management. This review repositions allergists as essential oncology partners, advancing irAE care from crisis response to precision immuno-toxicology.

## Graphical Abstract

**Figure:**
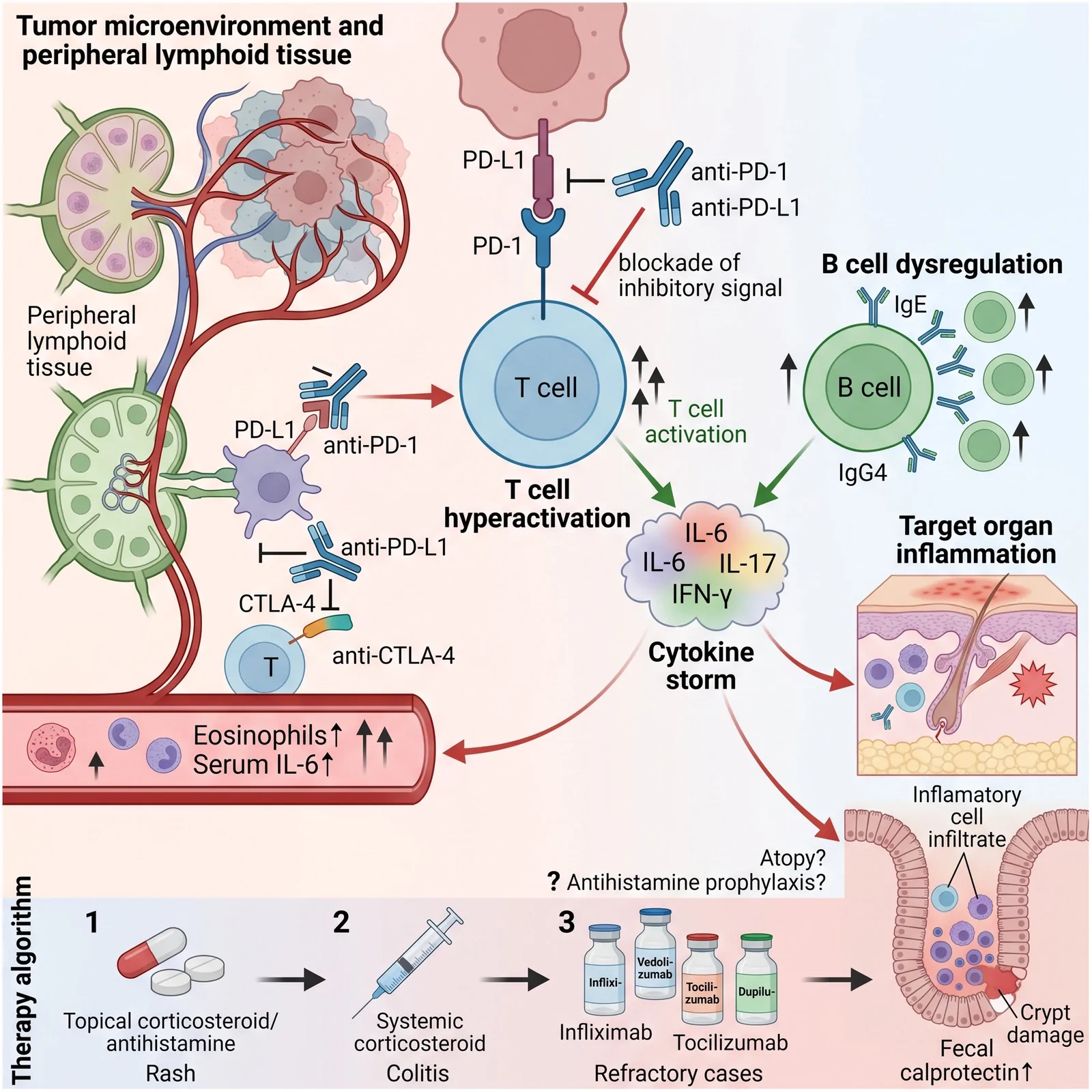

## Introduction: the allergist’s new frontier

The past decade has witnessed a paradigm shift in oncology with the advent of immune checkpoint inhibitors (ICIs). Agents targeting programmed cell death protein 1 (PD-1), its ligand PD-L1, and cytotoxic T-lymphocyte-associated protein 4 (CTLA-4) have become standard-of-care for over 15 cancer types, including melanoma, non-small cell lung cancer, renal cell carcinoma, and Hodgkin lymphoma (1–4). By blocking inhibitory receptors on T cells, ICIs unleash anti-tumor immunity. However, this same mechanism disrupts peripheral tolerance, triggering a distinct class of inflammatory toxicities known as immune-related adverse events (irAEs) (5–7).

While this review focuses specifically on cutaneous and gastrointestinal irAEs—the most prevalent and clinically challenging manifestations encountered by allergists—it is important to acknowledge that irAEs can affect nearly every organ system. Endocrine irAEs (thyroiditis, hypophysitis, and adrenal insufficiency) occur in 10–20% of patients and often require permanent hormone replacement (5, 8–10). Pneumonitis, though less common (2–5%), carries significant mortality and requires prompt recognition (11). Neurological irAEs (peripheral neuropathy, myasthenia gravis, encephalitis) (12–15), and rheumatological irAEs (inflammatory arthritis, myositis, sicca syndrome) are increasingly recognized and may present diagnostic challenges (16, 17). The allergist and immunologist should maintain a high index of suspicion for these manifestations and collaborate with appropriate organ specialists when they arise.

As, IrAEs affect nearly every organ system, with the skin (30-70% of patients) and gastrointestinal irAEs occur in 20-50% of patients, derived from prospective cohort studies, pooled estimates from clinical trials, meta-analysis, and systematic reviews (18, 19). For the practicing allergist and clinical immunologist, irAEs represent both a clinical challenge and a unique scientific opportunity. These iatrogenic inflammatory syndromes mimic classical allergic and autoimmune diseases, yet they arise from a fundamentally distinct immunological mechanism: the reversal of T-cell exhaustion rather than classic type I–IV hypersensitivity (20, 21).

As of 2025, over 50% of cancer patients receive ICIs either as monotherapy or in combination regimens. The allergist is increasingly consulted to differentiate irAEs from true drug allergies, manage refractory inflammatory toxicities, and determine the safety of ICI rechallenge following severe reactions (22). A growing body of evidence suggests that irAE development is paradoxically associated with improved overall survival, with hazard ratios ranging from 0.46 to 0.62 depending on the timing and type of toxicity. This toxicity-efficacy relationship underscores the importance of expert management that enables patients to remain on therapy while controlling inflammatory side effects. The demand for allergist consultation in oncology settings has increased substantially, with retrospective analyses showing a 3- to 5-fold increase in immunotherapy-related referrals to allergy/immunology services (23–27).

The goals of this review are to provide a mechanistic framework distinguishing irAEs from classical hypersensitivity, characterize the clinical spectrum of cutaneous and gastrointestinal irAEs with diagnostic pearls, present an evidence-based management algorithm incorporating emerging biologic therapies, address the role of pre-existing atopy and eosinophilia in irAE risk, and define the allergist’s role in rechallenge decisions.

## Methodology

This review was conducted as a narrative review employing systematic literature search principles to ensure comprehensive coverage of cutaneous and gastrointestinal immune-related adverse events (irAEs) associated with immune checkpoint inhibitors (ICIs). A comprehensive literature search was performed using PubMed/MEDLINE, Embase, Cochrane Library, and Web of Science for publications from January 2015 to December 2025, capturing both foundational studies and the most recent evidence. Search terms included combinations of “immune checkpoint inhibitors, “ “PD-1 inhibitor, “ “PD-L1 inhibitor, “ “CTLA-4 inhibitor” with “immune-related adverse events, “ “cutaneous toxicity, “ “colitis, “ “dermatologic toxicity, “ and “gastrointestinal toxicity.” Mechanism-focused terms included “pathophysiology, “ “T cell, “ “regulatory T cell, “ “cytokine, “ and “autoantibody, “ while management-focused terms included “corticosteroid, “ “biologic, “ “infliximab, “ “vedolizumab, “ “tocilizumab, “ “dupilumab, “ and “guidelines.” Only English-language publications were included in the final synthesis.

Studies were selected based on relevance to ICI-associated cutaneous or gastrointestinal irAEs, addressing pathophysiology, clinical presentation, diagnosis, or management. Included study types encompassed systematic reviews, meta-analyses, randomized controlled trials, prospective and retrospective cohort studies, case series (n≥5), relevant case reports for rare toxicities, and international guidelines from ASCO, ESMO, NCCN, and SITC. Priority was given to systematic reviews, meta-analyses, large cohort studies, and recent high-impact publications (2023–2025). Data extraction was performed by the primary author and independently verified by a second reviewer, with discrepancies resolved through consensus with the senior author. Evidence was synthesized narratively, organized thematically by pathophysiological mechanisms, clinical presentation, diagnostic approaches, and management algorithms. While formal GRADE or PRISMA methodology was not employed given the narrative scope, evidence quality was assessed implicitly with explicit acknowledgment of limitations including publication bias, selection bias, and heterogeneity across studies. The narrative approach was selected to integrate diverse evidence types—from basic immunology to clinical guidelines—and provide clinically actionable synthesis for the target audience of allergists and clinical immunologists.

To strengthen the evidence base, the review integrates recent meta-analyses and large cohort studies published between 2024 and 2025, including a systematic review and meta-analysis of peripheral blood cell counts as predictors of irAEs (60 studies, 16, 736 patients), a comparative meta-analysis of infliximab versus vedolizumab in ICI colitis, and a proportional meta-analysis of novel checkpoint inhibitors (LAG-3, TIGIT, TIM-3) from ASCO 2025. These additions, along with explicit acknowledgment of evidence limitations and ongoing clinical trials, ensure that the review provides a balanced, evidence-informed framework for clinical decision-making.

## Pathophysiology: from T-cell exhaustion to inflammatory dysregulation

### Normal immune checkpoint physiology

Under homeostatic conditions, immune checkpoints serve as critical brakes on T-cell activation. PD-1 is expressed on activated T cells, B cells, and myeloid cells. Engagement with its ligands (PD-L1 on antigen-presenting cells and tumor cells; PD-L2 on dendritic cells) transmits inhibitory signals that limit T-cell proliferation, cytokine production, and cytotoxicity (28). CTLA-4, expressed primarily on regulatory T cells (Tregs) and activated conventional T cells, outcompetes the co-stimulatory molecule CD28 for binding to CD80/CD86 on antigen-presenting cells, thereby raising the activation threshold for naïve T cells (29). In the tumor microenvironment, cancer cells exploit these checkpoints by upregulating PD-L1, inducing T-cell exhaustion and immune evasion (30). ICIs block this suppression, rejuvenating exhausted tumor-infiltrating lymphocytes (TILs) and enabling tumor destruction (**Figure 1A**).

**Figure 1:**
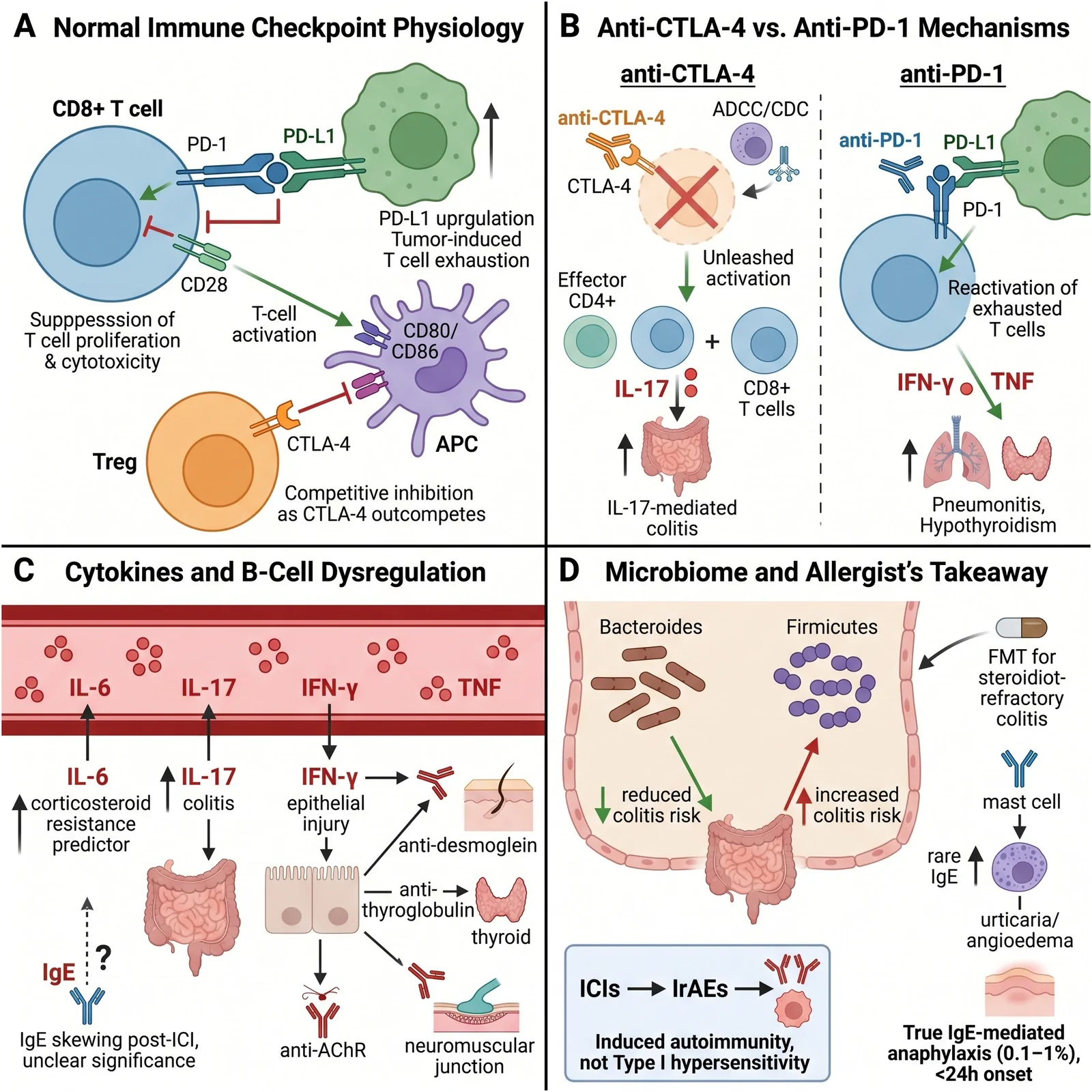
Pathophysiology of immune checkpoint inhibitor-induced irAEs. **(A)** Normal immune checkpoint physiology: PD-1/PD-L1 and CTLA-4/CD80/CD86 pathways maintain T-cell tolerance. **(B)** Divergent mechanisms: Anti-CTLA-4 depletes Tregs driving IL-17 colitis; anti-PD-1 reactivates CD8+ T cells driving Th1-mediated pneumonitis and hypothyroidism. **(C)** Cytokine dysregulation (IL-6, IL-17, IFN-γ) and B-cell autoantibody production characterize irAEs. **(D)** Gut microbiome composition modulates irAE risk, with FMT emerging as therapy. IrAEs represent induced autoimmunity, not Type I hypersensitivity, though rare IgE-mediated anaphylaxis (0.1–1%) requires exclusion (Image is created by Biorander and MS PowerPoint).

### Divergent mechanisms of irAE induction by anti-CTLA-4 versus anti-PD-1

Critically, anti-CTLA-4 and anti-PD-1 induce irAEs through distinct mechanisms, explaining their differential organ tropism and synergistic toxicity in combination therapy.

Anti-CTLA-4 (ipilimumab): CTLA-4 is constitutively expressed at high levels on regulatory T cells (Tregs) and is upregulated on conventional T cells upon activation. Importantly, CTLA-4 expression on Tregs is not uniform across tissues; Tregs in the colon, pituitary, and skin express particularly high levels of CTLA-4, which may explain the organ-specific predilection of anti-CTLA-4 toxicities. Ipilimumab binding to CTLA-4 depletes intratumoral and peripheral Tregs via antibody-dependent cellular cytotoxicity (ADCC) and complement-dependent cytotoxicity (CDC) (31). This Treg depletion, particularly in tissues with high baseline Treg activity, triggers unchecked effector T-cell activation. Additionally, anti-CTLA-4 enhances Th17 differentiation through both direct and indirect mechanisms. Directly, CTLA-4 blockade on CD4+ T cells promotes Th17 polarization by altering the TGF-β/IL-6 balance. Indirectly, the depletion of Tregs—which are major sources of TGF-β—reduces the suppressive signals that normally limit Th17 differentiation. This dual mechanism shifts the Treg/Th17 balance toward a pro-inflammatory Th17 phenotype, contributing to the predominance of IL-17-driven colitis (32, 33) (**Figure 1B**).

Anti-PD-1 (nivolumab, pembrolizumab): PD-1 blockade does not deplete Tregs to the same extent as anti-CTLA-4 therapy, as Tregs express lower levels of PD-1 compared to CTLA-4. However, it is important to note that Tregs do express PD-1, and PD-1+ Tregs exhibit enhanced suppressive functions in the tumor microenvironment. PD-1 blockade may actually increase the number and suppressive capacity of Tregs in some contexts, further distinguishing the mechanism from anti-CTLA-4 therapy (34, 35). This explains the higher incidence of pneumonitis and hypothyroidism with anti-PD-1 (Th1-mediated) compared to anti-CTLA-4, and the predominance of colitis with anti-CTLA-4 (Th17-mediated) (**Figure 1B**).

### Cytokine networks and B-cell dysregulation

IrAEs are characterized by profound cytokine dysregulation. Serum IL-6 levels correlate with irAE severity and predict corticosteroid resistance (36, 37). IL-17 is elevated in anti-CTLA-4 colitis but lack in anti-PD-1 colitis (38). IFN-γ drives keratinocyte apoptosis in cutaneous irAEs and intestinal epithelial injury in colitis (39, 40). Importantly, B cells also play a role. ICI therapy can induce autoantibody production, including anti-desmoglein antibodies (bullous pemphigoid), anti-thyroglobulin antibodies (thyroiditis), and anti-acetylcholine receptor antibodies (myasthenia gravis) (41). The frequency of autoantibody production varies by organ system; for instance, anti-BP180 antibodies are detected in 60–80% of ICI-induced bullous pemphigoid cases, while anti-thyroglobulin antibodies are found in approximately 30–50% of ICI-induced thyroiditis cases. The mechanistic role of these autoantibodies remains debated—whether they are causally involved in tissue damage or represent an epiphenomenon of immune activation. However, the correlation between antibody titers and disease severity in bullous pemphigoid suggests a pathogenic role in at least some irAEs (41).

Of particular interest is the role of IgG4 in ICI therapy. Elevated IgG4 levels have been associated with ipilimumab efficacy, and IgG4 subclass switching may represent a compensatory immunoregulatory response to checkpoint blockade. More recently, IgE skewing has been documented, with some patients developing elevated total IgE and drug-specific IgE following ICI initiation, though the clinical significance remains unclear (42, 43) (**Figure 1C**).

### The microbiome connection

The gut microbiome modulates irAE risk. Patients with *Bacteroides* dominance have lower rates of anti-CTLA-4 colitis, while those with *Firmicutes* enrichment have higher rates (44). Fecal microbiota transplantation (FMT) is emerging as a potential treatment for steroid-refractory ICI colitis (45). Key Takeaway for the Allergist: IrAEs are lacking “allergic reactions” to the ICI drug itself (Type I hypersensitivity) but rather an *induced autoimmunity* resulting from checkpoint blockade. However, true IgE-mediated anaphylaxis to ICIs can occur (incidence 0.1–1%) and should be excluded in patients presenting with urticaria or angioedema within 24 hours of infusion (46, 47) (**Figure 1D**).

## Cutaneous immune-related adverse events

Cutaneous irAEs are the earliest and most common toxicity, typically appearing within 3–6 weeks of ICI initiation (earlier with anti-CTLA-4, later with anti-PD-1) (48, 49).

### Maculopapular rash (most common)

The management of maculopapular rash follows a grade-based approach informed by major international guidelines, including ASCO, ESMO, and NCCN (50). For Grade 1 toxicity, guidelines generally support continuing ICI therapy while managing symptoms with emollients, oral antihistamines, and topical mid-potency corticosteroids. For Grade 2 toxicity, guidelines suggest that ICI continuation may be reasonable, though some experts recommend holding therapy if the rash does not improve rapidly with topical treatment; the addition of prednisone 0.5–1 mg/kg/day with a taper over 2 weeks is commonly employed (50).

For Grade 3 toxicity, guidelines recommend holding ICI therapy and initiating prednisone 1 mg/kg/day with a prolonged taper over 4–6 weeks, along with dermatology consultation. For Grade 4 toxicity, permanent discontinuation of ICI therapy is generally advised, with hospitalization for IV methylprednisolone 1–2 mg/kg/day; TNF inhibitors such as infliximab have been used in refractory cases, though evidence for this approach is limited to case reports (51–53).

Importantly, the strength of evidence supporting these recommendations varies considerably, with most guidance derived from expert consensus rather than randomized controlled trials. Clinicians should individualize management based on patient-specific factors and treatment response (51–53).

CTCAE Grading (version 5.0): Rash maculo-papular: Grade 1: <10% BSA with no symptoms or mild symptoms; Grade 2: 10–30% BSA with moderate symptoms; Grade 3: >30% BSA or severe symptoms limiting self-care ADL; Superinfection is not a grade criterion for this category.

Stevens-Johnson Syndrome/Toxic Epidermal Necrolysis (SJS/TEN): This is a separate diagnostic entity in CTCAE, not Grade 4 of rash. It is categorized under its own section with Grades 1-5 (Grade 3: SJS; Grade 4: SJS/TEN overlap; Grade 5: Death).

### Pruritus without rash

Pruritus occurs in 20–30% of ICI recipients, based on observational cohort studies and post-marketing surveillance data (54, 55). Mechanism involves IL-31 upregulation from activated T cells. Management: antihistamines (though less effective than in atopic dermatitis), gabapentin (100–300 mg TID) may be considered for neuropathic itch. Dose adjustments are required for elderly patients (starting dose 100 mg daily) and those with renal impairment (CrCl <60 mL/min). It is important to note that the evidence for gabapentin in ICI-related pruritus is primarily derived from case series and expert opinion, with no randomized controlled trials specifically addressing this indication. Moreover, aprepitant (80 mg daily for 5 days) has been reported in a limited number of case reports for refractory ICI-induced pruritus. This represents off-label use with very limited evidence and should not be considered a standard or recommended regimen; it may be considered only in highly refractory cases where standard therapies have failed and after careful risk-benefit discussion with the patient (56, 57).

### Bullous pemphigoid

Management of ICI-induced bullous pemphigoid is guided by the severity of skin involvement. For limited disease (Grade 1–2), several guidelines suggest that ICI therapy may be continued while initiating topical therapy with potent corticosteroids such as clobetasol, often in combination with doxycycline and niacinamide (58, 59). This approach is based on experience from spontaneous bullous pemphigoid, though prospective data in the ICI setting are lacking. For extensive disease (Grade 3–4), guidelines recommend holding ICI therapy and initiating systemic corticosteroids (prednisone 0.5–1 mg/kg/day) (50). A Cochrane review assessed 14 RCTs (1442 participants) for bullous pemphigoid treatments. The main treatment modalities evaluated were oral steroids, topical steroids, and doxycycline. The review concluded that doxycycline is less effective but causes less mortality than prednisolone for treating bullous pemphigoid (60–62). More recently, dupilumab has emerged as a promising steroid-sparing option based on retrospective case series. However, it is important to note that the evidence for these biologic agents is largely derived from case reports and small retrospective series, and no randomized controlled trials have been conducted specifically for ICI-induced bullous pemphigoid (63, 64). Preliminary evidence suggests dupilumab may be a promising steroid-sparing option for ICI-induced bullous pemphigoid, as it blocks IL-4/IL-13 signaling implicated in eosinophil recruitment. However, current evidence is limited to retrospective case series and case reports. A 2025 retrospective case series reported complete remission in 75% of patients, and a systematic review found dupilumab to be “highly effective” in most reported cases. It is critically important to note that dupilumab is FDA-approved for atopic dermatitis, asthma, and chronic rhinosinusitis with nasal polyposis, but NOT for bullous pemphigoid or ICI-induced dermatologic toxicities. Its use in this setting is entirely off-label and should be reserved for refractory cases after failure of standard therapies, with appropriate informed consent (65–68). Regarding prognosis, unlike spontaneous bullous pemphigoid, ICI-induced bullous pemphigoid may resolve after ICI discontinuation; however, approximately 40% of patients require ongoing immunosuppression (59, 69).

However, the incidence of bullous pemphigoid appears to vary by ICI regimen: anti-PD-1 monotherapy is associated with a higher incidence compared to anti-CTLA-4 monotherapy, though combination therapy may further increase risk (42). Furthermore, anti-BP180/BP230 ELISA antibody titers have been shown to correlate with disease severity in some studies, and monitoring antibody titers may guide treatment de-escalation, though prospective validation is needed (42, 59).

### Severe cutaneous adverse reactions

*SJS/TEN:* Rare (<0.1%) but life-threatening. Presents with prodromal fever, painful mucocutaneous lesions, and rapidly progressing epidermal detachment. Management: Immediate ICI discontinuation, hospitalization (burn unit), and multidisciplinary care. Recent large retrospective studies have questioned the efficacy of IVIG in SJS/TEN, with some showing no clear benefit. Cyclosporine (3–5 mg/kg/day) has shown more consistent benefit in some cohorts. TNF inhibitors (etanercept 50 mg subcutaneously weekly) have shown promise in early studies. The most critical intervention is prompt recognition, ICI cessation, and specialized burn/dermatology unit care, as mortality is significantly reduced in specialized settings (70–72).

*DRESS syndrome:* Fever, rash, eosinophilia, and internal organ involvement (liver, kidney, lung). Management: ICI discontinuation, systemic corticosteroids, and prolonged taper (DRESS has a median recovery of 6–8 weeks and can flare upon rapid steroid withdrawal) (73, 74).

### Other cutaneous manifestations

Vitiligo: Depigmented macules, particularly in melanoma patients. Immune correlate of anti-tumor response (associated with improved survival), no treatment needed (75). Psoriasiform dermatitis: Plaque psoriasis-like lesions, treat with topical steroids; avoid systemic methotrexate or cyclosporine which may blunt anti-tumor response (76). Lichenoid dermatitis: Violaceous flat-topped papules. Treat with topical or intralesional corticosteroids. Moreover, **Table 1** summarizes the key clinical outcomes, procedural approaches, and interventional data for immune-related adverse events based on the most current evidence available through 2025. These data encompass incidence rates, time to onset patterns, first-line corticosteroid responses, second-line biologic agent outcomes, and critical rechallenge data that inform decision-making for the allergist and clinical immunologist. The table emphasizes that while corticosteroids remain first-line therapy, biologic agents including dupilumab, infliximab, and vedolizumab have demonstrated efficacy in refractory cases. Notably, irAE development is paradoxically associated with improved overall survival, and rechallenge following toxicity resolution appears safe and effective for most patients, though combination ICI regimens carry higher recurrence risk.

**Table 1:** Clinical outcomes and management data for immune-related adverse events (irAEs).

Clinical parameter/outcome	Data/Finding	Procedure/intervention	Relevant details	Reference
Overall irAE Incidence	80.8% of patients experienced any-grade irAE	ICI administration (ipilimumab, nivolumab, pembrolizumab, atezolizumab, avelumab, durvalumab)	Highest incidence with ipilimumab + PD-1 inhibitor combination	(77)
Grade 3–4 irAE Incidence	8% of patients experience grade 3–5 irAEs	ICI therapy monitoring	45.9% in melanoma cohort; 30.4% in renal cell carcinoma cohort experienced grade 3–4 irAEs	(78, 79)
Time to irAE Onset	Variable by organ system	Serial clinical and laboratory monitoring	Colitis: 6–8 weeks (anti-CTLA-4), 12–16 weeks (anti-PD-1); Hepatitis: ~8–12 weeks; Endocrine: 9–18 weeks	Clinical consensus
First-Line irAE Management	Corticosteroids (88.2% of bullous pemphigoid cases)	Prednisone 0.5–2 mg/kg/day or IV methylprednisolone 1–2 mg/kg/day	Corticosteroids predominant treatment for any irAE	(65, 78)
Second-Line Biologic Agents	Infliximab, vedolizumab, tocilizumab, dupilumab, omalizumab	IV infusion or subcutaneous injection based on agent	For corticosteroid-refractory cases	(65, 80)
Biologic Response Data (Dupilumab for Bullous Pemphigoid)	Complete remission: 86/122 (70.5%); Partial remission: 29/122 (23.8%); No remission: 6/122 (4.9%)	Dupilumab 600 mg SC initial, then 300 mg Q2W	Average remission time: 5.7 months; Recurrence in 25/122 (20.5%)	(80)
ICI Rechallenge - Recurrence Rate	31.8% overall recurrence of same irAE (323/1016 cases)	ICI resumption after irAE resolution	Nephritis 50%, skin 44%, colitis 39% had highest recurrence	(79)
Rechallenge by ICI Regimen	Anti-PD(L)1: OR 0.70; Anti-CTLA-4: OR 0.88; Combination: OR 1.52	Same ICI rechallenge after irAE	Combination therapy associated with highest recurrence risk	(79)
Rechallenge Safety Comparison	Lower incidence of all-grade irAEs vs. initial treatment (OR 0.05, 95% CI 0.02-0.13)	ICI rechallenge	Also lower high-grade irAEs (OR 0.37, 95% CI 0.21-0.64)	(81)
Rechallenge Efficacy	Similar ORR (OR 0.69, 95% CI 0.39-1.20); Improved PFS (HR 0.56) and OS (HR 0.55) vs. permanent discontinuation	ICI rechallenge vs. permanent discontinuation	Rechallenge after PD as reason for initial stop: increased high-grade irAEs (OR 4.97)	(81)
Survival Association	irAE development associated with superior OS	ICI therapy	EO-irAE HR 0.622; ME-irAE HR 0.463 (vs. non-irAE reference 1.000)	(77, 82)
Specific irAE Survival Benefit	Hepatitis and hypophysitis most strongly associated with better survival	ICI (ipilimumab + nivolumab)	Survival benefit persisted for hepatitis (p=0.04) and hypophysitis (p=0.03) after adjusting for immortal time bias	(82)

ADCC, antibody-dependent cellular cytotoxicity; CDC, complement-dependent cytotoxicity; CI, confidence interval; CTCAE, Common Terminology Criteria for Adverse Events; EO-irAE, early-onset immune-related adverse event; HR, hazard ratio; ICI, immune checkpoint inhibitor; IV, intravenous; ME-irAE, medium-onset immune-related adverse event; OR, odds ratio; ORR, objective response rate; OS, overall survival; PD, progressive disease; PFS, progression-free survival; Q2W, every 2 weeks; SC, subcutaneous.

Data compiled from systematic reviews, meta-analyses, and large cohort studies published between 2020 and 2025. Incidence and outcome data reflect predominantly melanoma, non-small cell lung cancer, and renal cell carcinoma populations, as these represent the largest ICI-treated cohorts. Grade 3–4 irAEs are defined according to CTCAE version 5.0 criteria. Corticosteroid dosing should be individualized based on irAE severity, organ system involvement, and patient factors.

## Gastrointestinal immune-related adverse events

### ICI-induced colitis

The incidence of ICI-induced colitis varies significantly by regimen: anti-CTLA-4 monotherapy is associated with a 20–30% incidence, based on pooled data from phase 3 clinical trials and real-world cohorts (83). It is important to distinguish between clinical colitis (symptomatic diarrhea) and endoscopic colitis (inflammation detected on colonoscopy). Endoscopic colitis may be present in a significant proportion of patients without symptoms (subclinical colitis), with some studies reporting endoscopic abnormalities in up to 60% of patients receiving combination therapy. This distinction is clinically relevant because subclinical colitis does not typically require treatment interruption (83). The median time to onset differs as well, occurring at 6–8 weeks for anti-CTLA-4 and 12–16 weeks for anti-PD-1. Clinically, patients present with diarrhea, which may be watery or bloody, along with abdominal pain, urgency, and tenesmus. Constitutional symptoms such as fever and weight loss may occur in severe cases. Unlike infectious colitis, ICI colitis is characterized by relatively less prominent nausea or vomiting (84). However, nausea and vomiting are not uncommon, particularly when enteritis coexists. Prominent nausea or vomiting should prompt evaluation for concomitant enteritis, gastritis, or alternative etiologies (infectious, bowel obstruction, or tumor-related).

Endoscopic findings are regimen-dependent: anti-CTLA-4 colitis demonstrates ulcerations, friability, and granularity similar to Crohn’s disease, typically in a patchy distribution involving the left colon and rectum (85). Regarding the role of endoscopy, current guidelines do not recommend routine colonoscopy for all patients with suspected ICI colitis. Endoscopy should be considered in the following scenarios: (1) patients who are steroid-refractory, (2) cases with an unclear diagnosis (suspected infection or other etiologies), (3) patients with severe or atypical presentations, and (4) when histologic confirmation is needed to guide treatment decisions. In mild to moderate cases responding to corticosteroids, endoscopy may not be necessary (85).

In contrast, anti-PD-1 colitis is less severe, often showing normal-appearing mucosa with only microscopic inflammation. Histopathologically, anti-CTLA-4 colitis reveals neutrophilic cryptitis and crypt abscesses indistinguishable from inflammatory bowel disease (IBD), along with increased apoptosis and preservation of goblet cells (86). Anti-PD-1 colitis exhibits a lymphocytic or collagenous colitis pattern, with apoptosis more prominent than neutrophilic inflammation (86). The key differentiating feature from idiopathic IBD is that ICI colitis generally shows increased intraepithelial lymphocytes and epithelial apoptosis, whereas IBD typically demonstrates chronic architectural distortion including crypt branching and atrophy in most cases (87). However, this distinction is not absolute, and overlapping features may be present, particularly in patients with pre-existing IBD.

### Infectious colitis mimics, grading, and management algorithm

Before initiating immunosuppression for suspected ICI colitis, mandatory testing includes stool PCR for Clostridioides difficile, Campylobacter, Salmonella, Shigella, and E. coli O157:H7, as well as cytomegalovirus (CMV) PCR and histologic examination of biopsy specimens for CMV inclusions. While some guidelines recommend CMV evaluation primarily for steroid-refractory cases, it is prudent to consider CMV testing for all patients requiring immunosuppressive therapy, particularly those with risk factors for CMV reactivation (e.g., prior immunosuppression, organ transplantation, or lymphopenia) (88, 89). A critical red flag is that immunosuppression for ICI colitis can reactivate CMV or worsen C. difficile infection. Regarding grading and management, Grade 1 colitis is defined as ≤4 stools per day over baseline with no symptoms; management includes continuing ICI, administering loperamide 2–4 mg as needed (maximum 16 mg/day), oral hydration, and dietary modification with low-fiber, low-fat, and lactose-free foods (50). Grade 2 colitis involves 4–6 stools per day with mild abdominal pain and no interference with activities of daily living (ADLs); management typically involves holding ICI and initiating prednisone 0.5–1 mg/kg/day (starting at 40–60 mg daily), tapered over 4–6 weeks once symptoms resolve, with no role for empiric antibiotics. Budesonide 9 mg daily is less effective for Grade 2 or higher colitis (90, 91). Grade 3 colitis is defined as ≥7 stools per day, severe pain, fecal incontinence, interference with ADLs, and hospitalization indicated; management typically involves discontinuing ICI (permanent for anti-CTLA-4, though rechallenge with anti-PD-1 may be attempted after resolution), administering IV methylprednisolone 1–2 mg/kg/day, and converting to oral prednisone taper after 3–5 days of IV therapy (92).

Infliximab (5 mg/kg IV) may be considered as second-line therapy if no clinical response is observed by day 3–5 of IV steroids. Researchers suggests potential benefit, with reported response rates ranging from 32% to 85% across different studies (68, 93, 94). The standard approach is to assess clinical response following a single 5 mg/kg dose. If response is observed, a full induction regimen (weeks 0, 2, and 6) is generally recommended, followed by maintenance every 8 weeks if needed. If no response to the first dose, a second dose at week 2 may be considered, though lack of response to the initial dose should prompt consideration of alternative therapies. The wide variation in reported response rates reflects the heterogeneity of available evidence, which consists largely of retrospective cohort studies and case series rather than randomized controlled trials (95, 96).

Vedolizumab (300 mg IV at weeks 0, 2, and 6) may be considered for infliximab-refractory cases, particularly when endoscopic evidence suggests predominant lymphocyte trafficking. However, the evidence base remains limited. In addition to the initial case series by Bergqvist et al. (2017, n=28) and case reports by Zhang & Zheng (2022), more recent multicenter retrospective studies (2023-2025) have reported response rates of 60-70% in infliximab-refractory ICI colitis. However, these studies are limited by small sample sizes, retrospective design, and lack of comparator arms. A 2025 systematic review and meta-analysis suggested that vedolizumab may be associated with lower rates of colitis recurrence compared with infliximab, though this finding requires confirmation in prospective studies (67, 97, 98). Grade 4 colitis, characterized by life-threatening features including perforation, hemorrhage, or toxic megacolon, surgical consultation should be considered, infliximab 10 mg/kg, and consideration of fecal microbiota transplantation (FMT) in refractory cases (45, 99).

The management algorithms of cutaneous and gastrointestinal irAEs is guided by a stepwise, grade-based approach that aligns with the recommendations of major international guidelines, including those from the American Society of Clinical Oncology (ASCO), the European Society for Medical Oncology (ESMO), the National Comprehensive Cancer Network (NCCN), and the Society for Immunotherapy of Cancer (SITC). These guidelines converge on the principle of early recognition, accurate grading using the Common Terminology Criteria for Adverse Events (CTCAE), and timely intervention. For Grade 1 toxicities, all major guidelines concur that ICIs can be continued with close monitoring. For Grade 2 toxicities, guidelines generally recommend holding ICI therapy until symptoms improve to Grade ≤1, with symptomatic management using topical or systemic corticosteroids. For Grade 3 toxicities, guidelines generally recommend withholding ICI therapy and systemic corticosteroids. For Grade 4 life-threatening toxicities (e.g., SJS/TEN, toxic megacolon), ICI therapy permanent discontinuation is generally recommended, and patients require urgent hospitalization and multidisciplinary management. For corticosteroid-refractory cases, infliximab is recommended as second-line therapy for colitis, while vedolizumab is an alternative for infliximab-refractory cases (50).

It is critically important to note that none of the biologic agents discussed in this section are FDA-approved or EMA-approved specifically for the treatment of ICI-induced colitis. All recommended uses are off-label, extrapolated from their approved indications for inflammatory bowel disease, rheumatoid arthritis, or other immune-mediated conditions. The evidence supporting their use in ICI colitis consists primarily of retrospective cohort studies, case series, and expert opinion, with no randomized controlled trials specifically designed for this indication. Clinicians should discuss the off-label nature of these therapies with patients, obtain informed consent, and consider the risk-benefit profile in the context of each patient’s cancer status and overall health.

Hence, **Table 2** summarizes biologic options for corticosteroid-refractory ICI colitis, defined as no response after 3–5 days of high-dose IV methylprednisolone. Infliximab is the best-established second-line agent with rapid onset (24–72 hours) and 75–85% response rates. Vedolizumab offers a gut-selective alternative that preserves systemic anti-tumor immunity. Tocilizumab has promise in refractory cases given IL-6’s role in irAE pathogenesis, while ustekinumab has only limited anecdotal support. Biologic choice may be guided by prior therapy, organ involvement, and infection risk.

**Table 2:** Biologic agents for corticosteroid-refractory immune checkpoint inhibitor colitis.

Agent	Mechanism	Dosing	Response rate	Timing	Key evidence
Infliximab	TNF inhibitor	5 mg/kg IV week 0, 2, 6	75–85%	24–72 hours	(95, 96)
Vedolizumab	α4β7 integrin inhibitor	300 mg IV week 0, 2, 6	60–70%	1–2 weeks	(97, 98)
Tocilizumab	IL-6 receptor inhibitor	8 mg/kg IV monthly	50–60% (steroid-refractory)	48–72 hours	(100–102)
Ustekinumab	IL-12/23 inhibitor	90 mg subcut week 0, 4, then q8w	Limited data (anecdotal)	2–4 weeks	(103, 104)

IL, interleukin; IV, intravenous; JCO, Journal of Clinical Oncology; q8w, every 8 weeks; SC, subcutaneous; TNF, tumor necrosis factor. Refractoriness is defined as no clinical response after 3–5 days of IV methylprednisolone 1–2 mg/kg/day or recurrence upon tapering below prednisone 20 mg daily. Response rates reflect clinical improvement in diarrhea, abdominal pain, and endoscopic findings. Timing indicates median time to initial symptomatic improvement after first dose. All agents listed are used off-label for ICI colitis except where approved by local regulatory authorities. Response Rate’ definitions vary across studies: some report clinical response (symptomatic improvement in diarrhea and abdominal pain), while others report endoscopic response (mucosal healing on colonoscopy). Direct comparison of the 75-85% and 60-70% figures should be made with caution due to these definitional differences.

Regulatory Status: All agents listed are off-label for ICI colitis. FDA and EMA approvals exist for other indications (infliximab: IBD, rheumatoid arthritis; vedolizumab: IBD; tocilizumab: rheumatoid arthritis, cytokine release syndrome; ustekinumab: psoriasis, IBD, psoriatic arthritis). Clinicians should verify current regulatory status in their jurisdiction and obtain informed consent for off-label use.

It is important to acknowledge that the evidence supporting biologic therapy for steroid-refractory irAEs varies considerably in quality. Most recommendations are derived from: Level 1 evidence (RCTs): None specifically for ICI irAEs; data extrapolated from IBD and rheumatology trials, Level 2 evidence (Meta-analyses): Emerging for infliximab vs. vedolizumab in ICI colitis, Level 3 evidence (Prospective cohort studies): Limited; mostly single-center experiences, Level 4 evidence (Retrospective cohorts and case series): Majority of available data, Level 5 evidence (Expert opinion): Significant component of guideline recommendations. Ongoing Clinical Trials: NCT05543226: Montelukast for irAE prophylaxis in atopic patients (currently enrolling), NCT04823456: Cetirizine prophylaxis for cutaneous irAEs (completed), and several phase II trials of tocilizumab and rituximab for steroid-refractory irAEs are ongoing.

***Critical clinical point:*** Anti-TNF therapy for ICI colitis appear less to compromise anti-tumor efficacy (105, 106). A 2024 meta-analysis found no difference in progression-free survival between ICI colitis patients treated with infliximab versus steroids alone (107).

### ICI-induced enteritis (small bowel)

ICI-induced enteritis is less common than colitis but remains underrecognized in clinical practice. The incidence is not well-defined, though some studies suggest it occurs in 10-20% of patients with GI irAEs. It appears to be more common with anti-CTLA-4 and combination therapy compared to anti-PD-1 monotherapy. Patients present with nausea, vomiting, abdominal pain, and weight loss, notably without diarrhea (108, 109). Diagnosis is challenging due to the limited accessibility of the small bowel. CT enterography showing small bowel wall thickening is often the first imaging modality; capsule endoscopy can provide direct visualization but carries a risk of capsule retention (particularly in patients with known strictures or prior abdominal surgery). Management is similar to that of ICI colitis; however, budesonide 9 mg daily may be more effective for isolated small bowel enteritis compared to its limited efficacy in colitis (110, 111). When enteritis and colitis coexist, treatment typically follows the colitis management algorithm, as systemic corticosteroids and biologics (e.g., infliximab) address both sites of inflammation.

## The unique role of atopy and eosinophilia

### Pre-existing atopy as a risk factor

The relationship between pre-existing atopic conditions and the risk of irAEs is an area of active investigation, with evidence suggesting both potential associations and complexities. While some retrospective analyses have reported that patients with atopic dermatitis, asthma, or allergic rhinitis may have a higher incidence of cutaneous irAEs, other studies have not consistently confirmed this association, and and prospective data are lacking. It is important to explicitly state that most evidence is derived from retrospective cohorts, which carry inherent risk of bias and confounding. Prospective studies are urgently needed to confirm this association and establish the clinical utility of atopy as a predictive biomarker (112–115). Moreover, the role of atopy in cancer development and irAE susceptibility remains incompletely defined. The proposed mechanism involves baseline Th2 skewing; when PD-1 is blocked, Th2 cells may become reactivated, potentially leading to eosinophil recruitment and IgE production. However, this mechanistic hypothesis requires further validation, and the clinical significance of Th2 skewing in irAE pathogenesis remains uncertain. Importantly, a study specifically examining patients with asthma found that ICI therapy did not exacerbate their respiratory symptoms, suggesting that the impact of atopy may be organ-specific and not uniformly detrimental across all tissues. Therefore, while a history of atopy may be a consideration in risk assessment, it should not be viewed as a definitive or universal predictor of irAE development (112–115). It has also been reported prophylaxis has some potential but larger confirmatory studies are needed before routine prophylaxis can be recommended. The role of prophylactic antihistamines remains an area of ongoing investigation (116–118).

### Eosinophilia as a biomarker

ICI-induced eosinophilia, variably defined across studies with thresholds ranging from 300 to 500 cells/μL, occurs in a significant subset of patients. Given the variability in laboratory normal ranges (typically 300-500/μL), and the fact that mild eosinophilia (500-1, 500 cells/μL) may have multiple causes in cancer patients (including paraneoplastic phenomena, infections, or other medications), we recommend focusing on persistent or new-onset eosinophilia (>500 cells/μL) as a clinically relevant finding (119–122). However, the prognostic significance of eosinophilia remains controversial. While some earlier studies suggested a paradoxical association with improved overall survival, recent and more robust evidence, including a large meta-analysis, has challenged this finding. The mechanisms underlying this potential association are speculative but may include: (1) direct antitumor activity of eosinophils through degranulation and antibody-dependent cellular cytotoxicity; (2) eosinophilia as a marker of stronger overall immune activation (proxy for T-cell response); or (3) the association may simply reflect immortal time bias in observational studies (119–122).

For instance, a meta-analysis of patients with non-small cell lung cancer found that pretreatment eosinophilia was associated with poorer progression-free survival, underscoring the complexity and heterogeneity of eosinophils’ role in the tumor microenvironment and the variability across different cancer types and treatment regimens. Consequently, there is currently no consensus on the clinical utility of eosinophilia as a reliable prognostic biomarker, and its interpretation should be contextualized within the specific patient population and treatment setting (119–122). Regarding management, there are no established guidelines specifically for treating ICI-induced eosinophilia. Current expert opinion generally advises against treating asymptomatic eosinophilia. Eosinophilia itself does not typically require treatment. Current expert opinion advises against intervening solely to lower eosinophil counts. For patients with severe, refractory pruritus and marked eosinophilia, targeted therapies such as mepolizumab or dupilumab have been described in case reports. However, given the lack of robust clinical trial data and the unresolved questions about eosinophilia’s prognostic impact, such interventions should be considered experimental and reserved for individual cases after careful risk-benefit assessment. Treatment decisions should be individualized, and consultation with hematology or allergy/immunology specialists may be appropriate for complex cases (123–125).

### IgE and ICI therapy

Serum total IgE rises in 15–20% of immune checkpoint inhibitor recipients, based on retrospective analyses of clinical trial and real-world cohorts (63). However, total IgE can be nonspecifically elevated in cancer patients due to the tumor itself, cancer cachexia, or concomitant infections. It may be that significant elevations (e.g., >2 times the upper limit of normal) have higher predictive value for irAE development, though this has not been formally studied. Routine monitoring of IgE levels is not currently recommended outside of clinical investigation. Drug-specific IgE, such as anti-pembrolizumab IgE, has been detected in patients experiencing anaphylaxis but remains poorly available in clinical practice (126). Skin testing for ICI allergies has limited sensitivity and specificity; false negatives are relatively common, and standardized testing protocols have not been established. The negative predictive value of skin testing for ICI anaphylaxis is unknown. An important unanswered question is whether ICIs increase the risk of new food allergy or anaphylaxis; current data suggest no, but ongoing surveillance is necessary. Hence, the overall transition from the baseline atopic state to clinical outcomes is summarized in **Figure 2**.

**Figure 2:**
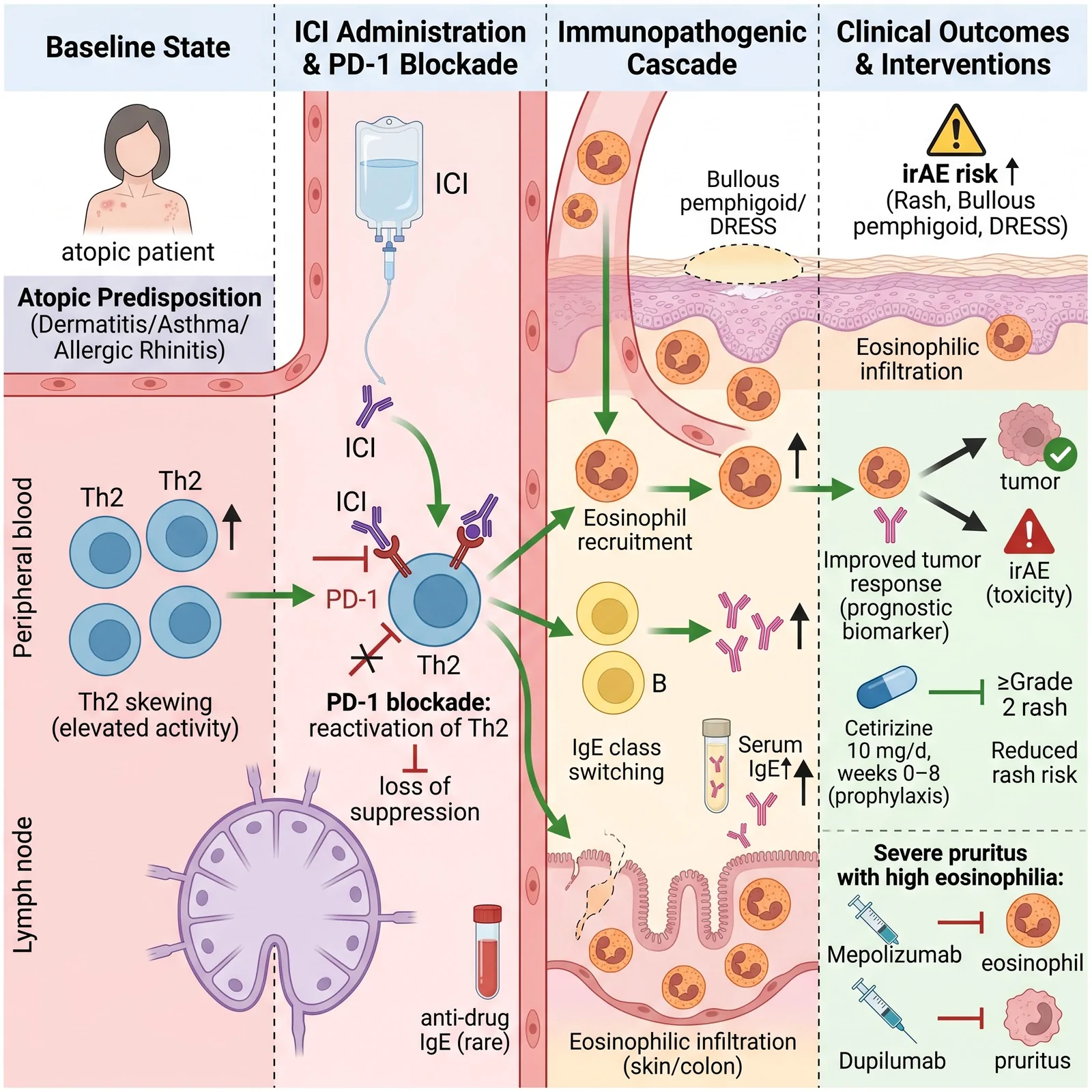
The role of atopy, eosinophilia, and ige in immune-related adverse events. This figure summarizes the unique contribution of atopic background and eosinophil biology to ICI toxicity. Pre-existing atopic dermatitis, asthma, or allergic rhinitis confers a 1.5- to 2-fold increased risk of cutaneous irAEs, driven by baseline Th2 skewing and PD-1 blockade-mediated reactivation of Th2 cells, leading to eosinophil recruitment and IgE production. Prophylactic cetirizine 10 mg daily for 8 weeks in atopic patients reduced grade ≥2 rash from 40% to 15% (p=0.03) in a small randomized trial. ICI-induced eosinophilia (>500 cells/μL) occurs in 20–30% of patients, based on retrospective cohort studies and case series, and correlates with cutaneous irAEs (bullous pemphigoid, DRESS), eosinophilic colitis, and paradoxically improved overall survival. Symptomatic eosinophilia with severe pruritus (>5, 000 cells/μL) may be treated with mepolizumab or dupilumab. Serum total IgE rises in 15–20% of ICI recipients, particularly those with bullous pemphigoid or DRESS. Drug-specific IgE has been detected in anaphylaxis cases but is poorly available. ICIs poorly clear increase new food allergy risk. Key takeaway: Atopy increases irAE risk, eosinophilia predicts both toxicity and favorable tumor response, and prophylactic antihistamines may benefit high-risk patients (Image is created by Biorander and MS PowerPoint).

## Diagnostic approach: algorithm for the allergist

When a patient on immune checkpoint inhibitor therapy presents with new-onset rash or diarrhea, the first and most critical step for the allergist is to determine the timing of symptom onset relative to the most recent ICI infusion. This temporal distinction guides the entire diagnostic workup, as immediate reactions occurring within 24 hours raise concern for true IgE-mediated drug allergy, whereas later presentations are more consistent with immune-related adverse events representing induced autoimmunity rather than classical hypersensitivity as illustrated in **Figure 3**.

**Figure 3:**
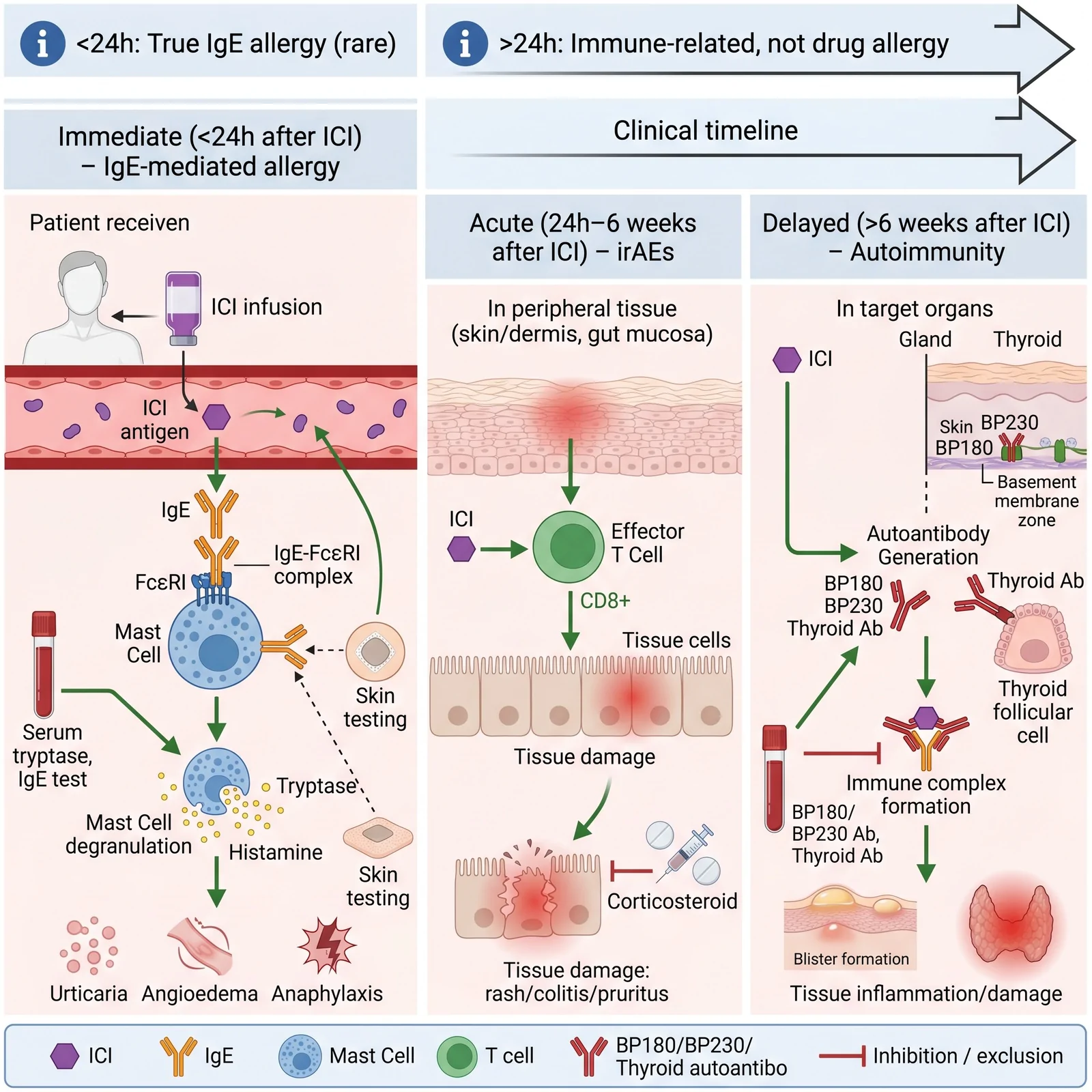
Diagnostic algorithm for evaluating suspected immune checkpoint inhibitor reaction. This algorithm provides a stepwise framework for the allergist when a patient on ICI therapy presents with new-onset rash or diarrhea. The critical branching point is timing of symptom onset relative to the most recent ICI infusion. For reactions occurring within 24 hours, suspect urticaria, angioedema, or anaphylaxis; this warrants evaluation for true Type I hypersensitivity including serum tryptase, IgE levels, and skin testing. For reactions occurring between 24 hours and 6 weeks, suspect maculopapular rash, colitis, or pruritus; these represent likely acute irAEs (induced autoimmunity) and should be managed with topical or systemic corticosteroids based on CTCAE grade. For reactions occurring after 6 weeks, suspect bullous pemphigoid, sicca syndrome, or thyroiditis; these represent likely delayed irAEs and should prompt specific autoantibody testing including anti-BP180/BP230 for bullous pemphigoid or thyroid antibodies for thyroiditis. Key clinical pearl: Immediate reactions (<24 hours) suggest rare true IgE-mediated drug allergy (0.1–1%). It is important to distinguish between: (1) reactions to the first infusion (more likely true allergy) and (2) reactions to subsequent infusions, which may represent accelerated reactions or infusion-related reactions (cytokine release syndrome-like). The algorithm should distinguish anaphylaxis (hypotension, wheezing, stridor, angioedema) from infusion-related reactions (fever, chills, mild rash without respiratory compromise). Fever assessment: In patients presenting with fever, consider: (1) infection (bacterial, viral, fungal), (2) irAE (e.g., colitis, pneumonitis, hepatitis), (3) tumor fever, or (4) infusion reaction. Prompt evaluation for infection with blood cultures, chest imaging, and urine analysis is essential before attributing fever to irAE. This diagnostic algorithm should be integrated with CTCAE grading to form a two-dimensional ‘time-grade’ decision framework: Early onset (<24 hours) with severe symptoms → evaluate for anaphylaxis; late onset (>6 weeks) with any grade → evaluate for autoimmune phenomena; intermediate onset with Grade 3–4 severity → urgent irAE management (Image is created by Biorander and MS PowerPoint).

## Management of corticosteroid-refractory irAEs

### Defining steroid refractoriness

No response after 3–5 days of IV methylprednisolone 1–2 mg/kg/day, or recurrence upon tapering below 20 mg prednisone daily (127). It should be noted that the appropriate observation period may vary by organ system: for hepatitis, a longer observation period (5–7 days) may be appropriate before declaring steroid-refractoriness, whereas for severe colitis, second-line therapy should be initiated if there is no response after 3 days of high-dose steroids.

### Biologic step-up protocol (off-label use)

For patients with corticosteroid-refractory ICI colitis, several biologic agents may be considered, though all are used off-label for this indication. The choice of agent should be individualized based on prior therapies, organ involvement, and patient-specific factors.

First-line (steroid-refractory, preferred): Infliximab 5 mg/kg IV at weeks 0, 2, and 6. Alternative first-line option (gut-selective): Vedolizumab 300 mg IV at weeks 0, 2, and 6, then every 8 weeks. The choice between infliximab and vedolizumab should be individualized. Currently, most guidelines recommend infliximab as the preferred first-line biologic based on more extensive evidence, while vedolizumab is often reserved for infliximab-refractory cases or when gut selectivity is particularly desired (104, 128–131).

Infliximab is FDA-approved for Crohn’s disease, ulcerative colitis, rheumatoid arthritis, and other inflammatory conditions, but not specifically for ICI colitis. Its use in this setting is supported by observational studies and expert consensus (104, 128). Monitor for infusion reactions; Routine premedication (antihistamines + acetaminophen ± corticosteroids) is not routinely required but may reduce the risk of infusion reactions in patients with prior hypersensitivity; however, premedication may mask early signs of infusion reactions. The decision to premedicate should be individualized based on prior reaction history and clinical context. Second-line (if infliximab fails or is generally not recommended): Vedolizumab 300 mg at weeks 0, 2, and 6, then every 8 weeks. Vedolizumab is FDA-approved for moderate-to-severe ulcerative colitis and Crohn’s disease but not for ICI colitis. Its gut-selective mechanism preserves systemic anti-tumor immunity, which is a theoretical advantage over infliximab, though comparative effectiveness data are lacking (129–131). Third-line (limited data/refractory cases): Options with emerging evidence include ustekinumab (∼6 mg/kg IV then 90 mg SC) and tocilizumab (8 mg/kg monthly). Both are used off-label for ICI colitis. Ustekinumab is FDA-approved for psoriasis, psoriatic arthritis, and IBD, while tocilizumab is approved for rheumatoid arthritis and cytokine release syndrome. Tocilizumab has shown promise in small series for concomitant steroid-refractory colitis and arthritis but is not standard of care (132–134). It is important to distinguish the evidence by organ system: tocilizumab has stronger evidence in immune-related arthritis and hepatitis (where IL-6 plays a prominent pathogenic role) compared to colitis. None of these agents have regulatory approval specifically for irAE management but in investigational status.

***Emerging therapies:*** JAK inhibitors (tofacitinib 10 mg BID): Case reports suggest efficacy, but concern for increased infection and thrombosis. The FDA has issued a black box warning for tofacitinib regarding increased risk of serious infections, malignancy, and thrombosis. Its use in cancer patients requires extreme caution and should only be considered in highly refractory cases with multidisciplinary input. Not FDA-approved for irAEs (135, 136). Fecal microbiota transplantation (FMT): Single-center study (n=15) showed 80% response rate in steroid-refractory ICI colitis, with associated increase in *Bacteroides* abundance. The evidence for FMT in ICI colitis is limited to case reports and small series, despite its promise, there are currently no large-scale prospective studies, and the optimal donor selection, preparation, and administration route remain undefined. FMT should be considered experimental and reserved for highly refractory cases (99, 137).

### Management of infectious complications during immunosuppression

Prophylaxis for patients on prednisone ≥20 mg/day × ≥4 weeks: *Pneumocystis jirovecii:* Trimethoprim-sulfamethoxazole 1 DS daily or atovaquone 1, 500 mg daily (alternative; expensive; must be taken with food for optimal bioavailability). Dose adjustments are required for renal impairment (CrCl <30 mL/min) (138, 139). *Herpes zoster:* Recombinant zoster vaccine (Shingrix) before ICI initiation. If already immunosuppressed, acyclovir 400 mg BID prophylaxis or valacyclovir 500 mg daily (alternative, but less studied for prophylaxis in this setting) (140).

## The allergist’s role in ICI rechallenge

### Decision framework

Rechallenge with the same immune checkpoint inhibitor following a severe immune-related adverse event is a high-stakes decision that balances anti-tumor efficacy against the risk of toxicity recurrence. The general principles for rechallenge are as follows: For Grade 1 irAE, continue ICI without interruption. For Grade 2 irAE (except colitis), hold ICI until resolution to Grade ≤1, then rechallenge. For Grade 2 colitis, expert consensus generally recommends permanent cessation of anti-CTLA-4, though rechallenge with anti-PD-1 may be considered on a case-by-case basis. For Grade 3 rash (excluding SJS/TEN), rechallenge may be attempted after resolution. This decision should be made following discussion by a multidisciplinary tumor board (MDT) involving oncology, allergy/immunology, and relevant organ specialists, rather than by a single specialist alone. For Grade 3 colitis, most guidelines recommend permanent cessation of anti-CTLA-4; rechallenge with anti-PD-1 may be cautiously considered only if tumor response is robust and no alternative therapy exists. Grade 4 irAE of any type: Permanent discontinuation of all ICIs is generally recommended. However, isolated case reports have described successful rechallenges following recovery from Grade 4 visceral irAEs (e.g., myocarditis, hepatitis, nephritis) in patients with exceptional tumor responses and no alternative therapeutic options. Such individual exceptions require individualized MDT decision-making, weighing tumor burden, available alternatives, and the patient’s preferences. It is important to note that these recommendations are based primarily on expert opinion and observational data rather than high-level evidence (141).

### Desensitization for confirmed ICI allergy

For the rare patient with confirmed IgE-mediated anaphylaxis to an ICI, desensitization protocols are available. This protocol applies only to confirmed IgE-mediated allergies and is not applicable to irAE rechallenge (which represents a distinct phenomenon of induced autoimmunity). An example protocol for nivolumab includes six steps: Step 1 involves 1:100 dilution at 1 mL/hour for 30 minutes; Step 2 involves 1:10 dilution at 1 mL/hour for 30 minutes; Step 3 involves 1:10 dilution at 5 mL/hour for 30 minutes; Step 4 involves 1:10 dilution at 10 mL/hour for 30 minutes; Step 5 involves full concentration at 10 mL/hour for 30 minutes; and Step 6 involves full concentration at 20 mL/hour for the remainder of the infusion. Premedication should include cetirizine 10 mg, montelukast 10 mg, and dexamethasone 10 mg IV (46, 142). It is important to note that the infusion rate units (mL/hour) must be specified for the standard concentration of the drug (e.g., nivolumab 10 mg/mL). Readers should consult the original protocol for complete details.

### Switching between ICIs

Regarding switching between ICIs after an irAE from one ICI class, the following principles apply: Switching from anti-CTLA-4 to anti-PD-1 is generally safe, with a recurrence rate of any irAE of approximately 30% (143, 144). Switching from anti-PD-1 to a different anti-PD-1 agent is safe if the original irAE has resolved to Grade ≤1 in the absence of prior anaphylaxis. However, the risk of cross-reactivity between different anti-PD-1 antibodies (e.g., pembrolizumab vs. nivolumab) is not well-characterized. In the event of an allergic reaction, sensitization to excipients such as polysorbate 80—which is present in multiple ICI formulations—should be considered. However, switching from anti-CTLA-4 to a different anti-CTLA-4 agent is less or no recommended after Grade ≥3 toxicity, as the recurrence rate exceeds 50% (145–147).

Based on the diagnostic principles outlined above and the grade-based management strategies for each toxicity, we have developed an integrated algorithm that guides clinicians from initial presentation through diagnosis, grading, management, and rechallenge decision-making. This unified framework, presented in **Figure 4**, is designed as a practical reference tool for the allergist and clinical immunologist managing ICI-associated cutaneous and gastrointestinal irAEs.

**Figure 4:**
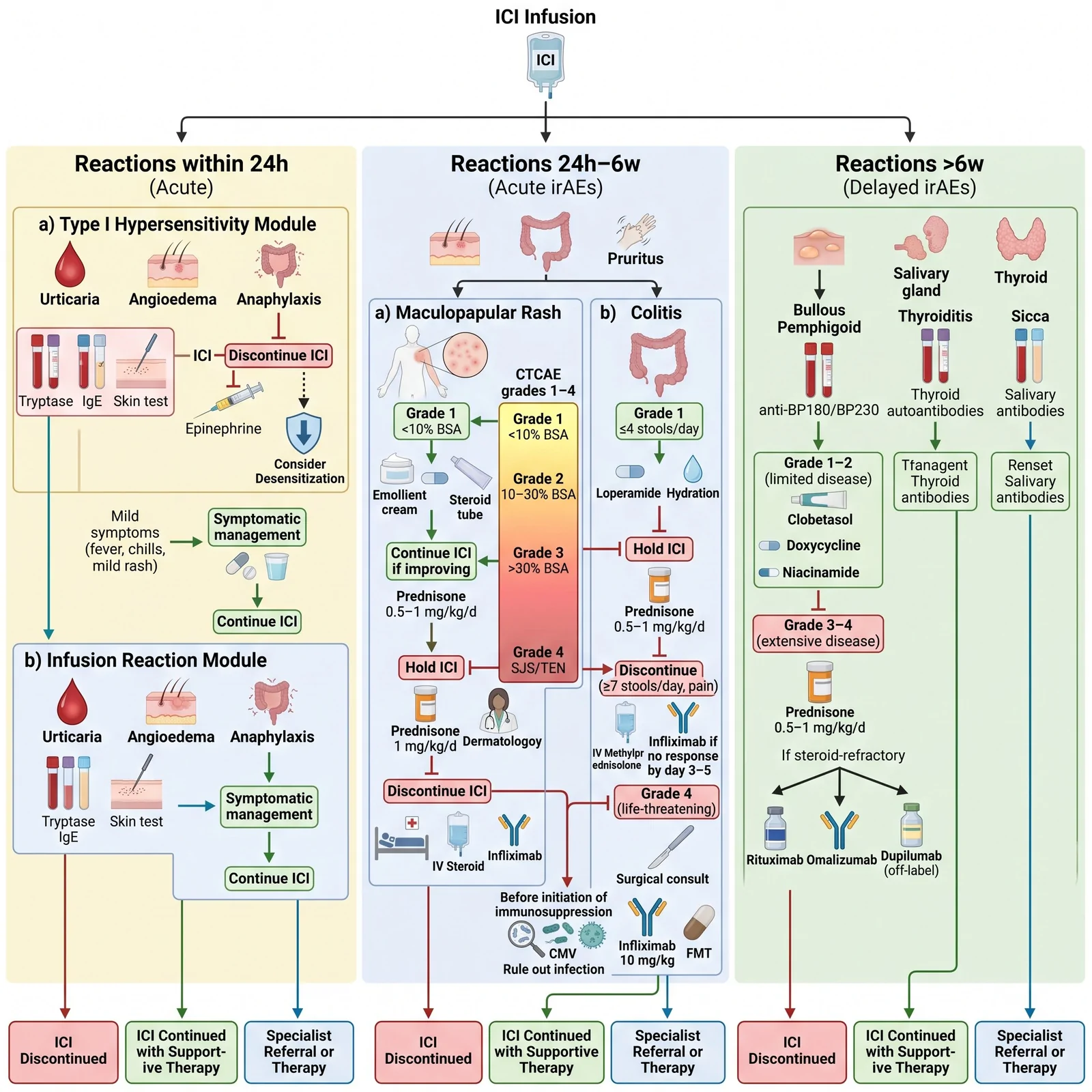
Integrated diagnostic and management algorithm for ICI-associated cutaneous and gastrointestinal irAEs. This algorithm provides a comprehensive stepwise framework for the allergist and clinical immunologist when a patient on immune checkpoint inhibitor (ICI) therapy presents with new-onset rash or diarrhea. The algorithm integrates three key dimensions: (1) timing of symptom onset relative to the most recent ICI infusion—a critical branching point that distinguishes immediate hypersensitivity reactions (<24 hours) from acute irAEs (24 hours to 6 weeks) and delayed irAEs (>6 weeks); (2) CTCAE (Common Terminology Criteria for Adverse Events) grading for each toxicity type; and (3) grade-based management strategies for maculopapular rash, bullous pemphigoid, and ICI-induced colitis. For immediate reactions (<24 hours), the algorithm distinguishes anaphylaxis (hypotension, wheezing, respiratory compromise) from infusion-related reactions (fever, chills, mild rash without respiratory compromise) and provides appropriate diagnostic workup and management pathways. For acute irAEs (24 hours to 6 weeks), CTCAE grading guides management from Grade 1 (continue ICI with symptomatic therapy) through Grade 4 (permanent ICI discontinuation, hospitalization). For delayed irAEs (>6 weeks), autoantibody testing directs organ-specific management. A fever assessment node is included, emphasizing the need to rule out infection before attributing fever to irAE. The algorithm concludes with a rechallenge decision framework, with multidisciplinary team (MDT) involvement recommended for complex decisions (Grade 3 colitis with anti-PD-1 rechallenge, Grade 4 exceptions). The algorithm is intended as a practical reference and should be used in conjunction with clinical judgment, international guidelines (ASCO, ESMO, NCCN, SITC), and multidisciplinary input. Color coding: green = continue/proceed with ICI; yellow = caution/consider holding ICI; red = stop ICI/urgent action; blue = diagnostic/workup steps; purple = rechallenge/decision-making (Image is created by Biorander and MS PowerPoint).

## Knowledge gaps and future directions

### Unanswered clinical questions

Prophylaxis: Do antihistamines or leukotriene inhibitors reduce irAE incidence in atopic patients? A phase 2 trial of montelukast 10 mg daily is ongoing (NCT05543226) —this trial is currently enrolling as of 2025. Biomarkers: Can a baseline cytokine panel predict irAE risk? Preliminary data suggest low IL-10 and high CXCL9 predict severe toxicity in melanoma cohorts; these findings require validation in larger, pan-cancer studies (148). Optimal biologic sequencing: Infliximab vs. vedolizumab as first-line for steroid-refractory colitis—are there predictive markers? Long-term outcomes: Do patients who receive biologics for irAEs have different cancer outcomes? Some retrospective data are now available: a 2025 study found no difference in overall survival among patients receiving dupilumab for cutaneous irAEs compared to matched controls, suggesting that this approach may be safe in the long term. However, prospective data are still needed (149–153).

### Emerging therapeutic concepts

Low-dose IL-2 therapy: Expands Tregs, potentially preventing or treating irAEs without blunting anti-tumor immunity. However, there are significant oncological concerns: expanding Tregs could theoretically weaken anti-tumor immunity and compromise the efficacy of ICIs. More data are needed to support the safety of this approach, particularly regarding the risk of tumor progression. Early-phase trials are underway but have not yet reported outcome (154). TLR antagonists: Blocking innate immune activation may prevent irAE initiation (155). Organ-specific homing inhibitors: Vedolizumab for gut; anti-CCR4 for skin (118, 156).

## Unresolved controversies in irAE management

Despite significant advances in understanding and managing immune-related adverse events, several critical controversies remain unresolved and warrant explicit acknowledgment. These controversies highlight the limitations of current evidence and underscore the need for continued investigation.

### The true role of pre-existing atopy

While several retrospective studies have suggested that pre-existing atopic conditions (asthma, atopic dermatitis, allergic rhinitis) confer an increased risk of cutaneous irAEs, this association has not been consistently replicated across all cohorts. Some studies have found no significant association, while others have suggested that atopy may actually be protective against certain irAEs. The mechanistic hypothesis involving Th2 skewing and PD-1 blockade-mediated reactivation remains plausible but unproven. Furthermore, the clinical significance of this association—whether it should influence ICI selection, dosing, or prophylactic interventions—remains highly uncertain. Until prospective, adequately powered studies are conducted, the role of pre-existing atopy in irAE risk stratification should be considered preliminary rather than established.

### The prognostic significance of eosinophilia

The prognostic value of ICI-induced eosinophilia represents one of the most contentious areas in irAE research. Early studies suggested a paradoxical association with improved overall survival, leading to the concept of eosinophilia as a favorable prognostic marker. However, more recent and methodologically robust evidence, including large meta-analyses, has challenged this finding. Some studies have reported that eosinophilia is associated with poorer progression-free survival, particularly in non-small cell lung cancer. These conflicting results likely reflect heterogeneity in patient populations, cancer types, treatment regimens, and the definition of eosinophilia itself. The field lacks consensus on whether eosinophilia should be considered a biomarker of toxicity, efficacy, both, or neither, and no guidelines exist for its clinical interpretation.

### Optimal biologic sequencing

The optimal sequencing of biologic agents for corticosteroid-refractory irAEs remains undefined. While infliximab is traditionally used as first-line biologic therapy for refractory colitis, emerging data suggest that vedolizumab may have comparable efficacy with a more favorable safety profile. However, no head-to-head randomized controlled trials have compared these agents specifically in the ICI colitis setting. The choice between infliximab and vedolizumab is further complicated by concerns that infliximab (a systemic TNF inhibitor) might theoretically compromise anti-tumor immunity, though current evidence does not support this concern. Conversely, vedolizumab’s gut selectivity may be advantageous but limits its utility for extraintestinal manifestations. The optimal timing of biologic initiation—whether to wait for corticosteroid failure or intervene earlier—also remains uncertain.

### The microbiome as a therapeutic target

While observational studies have consistently demonstrated associations between gut microbiome composition and irAE risk, translating these findings into therapeutic interventions has proven challenging. Fecal microbiota transplantation (FMT) has shown promise in small case series, but the optimal donor selection, preparation, dosing, and route of administration have not been established. Furthermore, the durability of FMT effects and the potential for long-term adverse consequences remain unknown. Whether microbiome modulation should be considered a standard therapeutic option or remains an experimental approach is a matter of ongoing debate.

### Rechallenge decision-making

Despite the availability of consensus guidelines, rechallenge decisions remain highly individualized and controversial. The evidence base for rechallenge recommendations is primarily derived from retrospective cohorts, with significant selection bias and confounding by indication. The recurrence rates reported in the literature vary widely (30–50%), and the factors predicting recurrence are not well-defined. Furthermore, the optimal duration of observation before rechallenge, the role of prophylactic immunosuppression during rechallenge, and the management of patients with incomplete irAE resolution remain unresolved. The decision to rechallenge ultimately depends on multiple patient-specific, tumor-specific, and treatment-specific factors, and there is no validated risk stratification tool to guide this decision.

### Prophylactic interventions

The role of prophylactic interventions to prevent irAEs remains controversial. While one small randomized trial suggested benefit of prophylactic cetirizine in atopic patients, this finding requires confirmation in larger, multicenter studies. The use of prophylactic corticosteroids, antihistamines, or other immunomodulators is not recommended by current guidelines due to concerns about blunting anti-tumor efficacy. Whether certain patient subgroups (e.g., those with autoimmune diseases, atopic conditions, or specific genetic polymorphisms) might benefit from prophylaxis while maintaining anti-tumor immunity is an area of active investigation.

### Long-term consequences of IrAEs

The long-term consequences of irAEs and their treatments remain poorly characterized. While short-term outcomes are well-described, the impact of irAEs on quality of life, organ function, and cancer survival beyond the first year of therapy is not well understood. Similarly, the long-term safety of biologic agents used to treat irAEs—including the risk of secondary malignancies, opportunistic infections, and autoimmune diseases—requires ongoing surveillance.

### Future directions – personalized risk stratification, predictive biomarkers, combination therapies

#### Personalized risk stratification

The development of multi-modal risk prediction models integrating clinical, laboratory, and genomic data represents a major unmet need. Recent advances include: Composite cytokine risk scores: A 2025 study developed a composite cytokine risk score integrating IL-7, IL-1RA, and CXCL-13, which independently predicted irAE in both univariable and multivariable analyses. Machine learning approaches: Multi-modal machine learning models incorporating blood-based biomarkers have shown promise in estimating severe irAE risk prior to ICI initiation in real-world cohorts. Novel biomarkers: Low triglyceride levels have been associated with increased risk of irAEs, suggesting that specific triglyceride species may serve as early biomarkers for irAE risk, particularly in female patients.

#### Predictive biomarkers

Beyond cytokines and blood counts, emerging biomarkers include: genetic polymorphisms: HLA alleles and other germline variants may predict irAE susceptibility; gut microbiome composition: Bacteroides/Firmicutes ratio as a potential predictor; and autoantibody profiles: Pre-existing autoantibodies may predict specific irAE types.

#### Combination therapies

As ICIs are increasingly used in combination—with each other, with chemotherapy, and with targeted therapies—the spectrum and severity of irAEs may change. Novel combinations incorporating LAG-3, TIGIT, and TIM-3 inhibitors are entering clinical practice with potentially distinct irAE profiles. Future research should focus on characterizing these profiles and developing tailored management algorithms.

## Conclusion

Immune checkpoint inhibitors have transformed oncology but have simultaneously created a new class of inflammatory diseases that fall squarely within the domain of allergy and immunology. Cutaneous and gastrointestinal irAEs are the most common and clinically impactful toxicities, yet they remain underrecognized and suboptimally managed outside of specialized centers. For the practicing allergist, five principles emerge from this review: Recognize the mechanism: IrAEs are not common IgE-mediated drug allergies (with rare exception). They represent checkpoint blockade-induced activation of self-reactive T and B cells. Grade before treating: CTCAE grading guides management. Grade 1 can usually continue ICI with symptomatic therapy. Grade 3–4 generally requires ICI hold and systemic immunosuppression. Biologics are safe and effective: Infliximab, vedolizumab, and tocilizumab are not just rescue therapies, they should be deployed early in steroid-refractory cases without fear of compromising anti-tumor immunity. Atopy and eosinophilia matter: Pre-existing atopy increases irAE risk. Eosinophilia is a biomarker for cutaneous toxicity and better prognosis. Rechallenge is nuanced: Permanent discontinuation is generally recommended for grade 4 irAEs and anti-CTLA-4 grade 3 colitis, but many patients can safely resume therapy after toxicity resolution. The allergist is no longer a passive consultant for drug reactions but an active partner in the oncology care team, enabling patients to remain on life-saving cancer immunotherapy while managing its inflammatory consequences.

Looking forward, the immunotherapy landscape is rapidly evolving with the development of next-generation immune checkpoint inhibitors targeting novel pathways including LAG-3 (lymphocyte activation gene-3), TIGIT (T-cell immunoglobulin and ITIM domain), and TIM-3 (T-cell immunoglobulin and mucin domain-3). These agents are currently being evaluated in clinical trials, both as monotherapy and in combination with established PD-1/PD-L1 inhibitors. Preliminary data suggest that LAG-3 inhibition may have a relatively favorable safety profile with lower rates of severe irAEs (approximately 10-15% grade ≥3) compared to CTLA-4 blockade, though this is based on early-phase trials. TIM-3 inhibitors may have a distinct pattern of toxicity with potential for increased immune-related skin reactions. TIGIT inhibitors appear to have a safety profile similar to PD-1 inhibitors, though combination regimens may potentiate toxicity. However, head-to-head comparisons are lacking, and the full spectrum of irAEs for these agents will only become clear with larger phase 3 trials and post-marketing surveillance. The allergist and immunologist will need to remain vigilant as these agents enter clinical practice, adapting management strategies to new toxicity spectra while continuing to apply the mechanistic principles established with current ICIs. Future research should focus on characterizing the unique irAE profiles of these novel agents and developing tailored management algorithms. As the ICI footprint expands into adjuvant and neoadjuvant settings, and as novel checkpoints enter the clinic, the demand for allergist expertise in immuno-toxicology will only grow. A visual summary of these mechanisms and management principles is provided in **Figure 5**.

**Figure 5:**
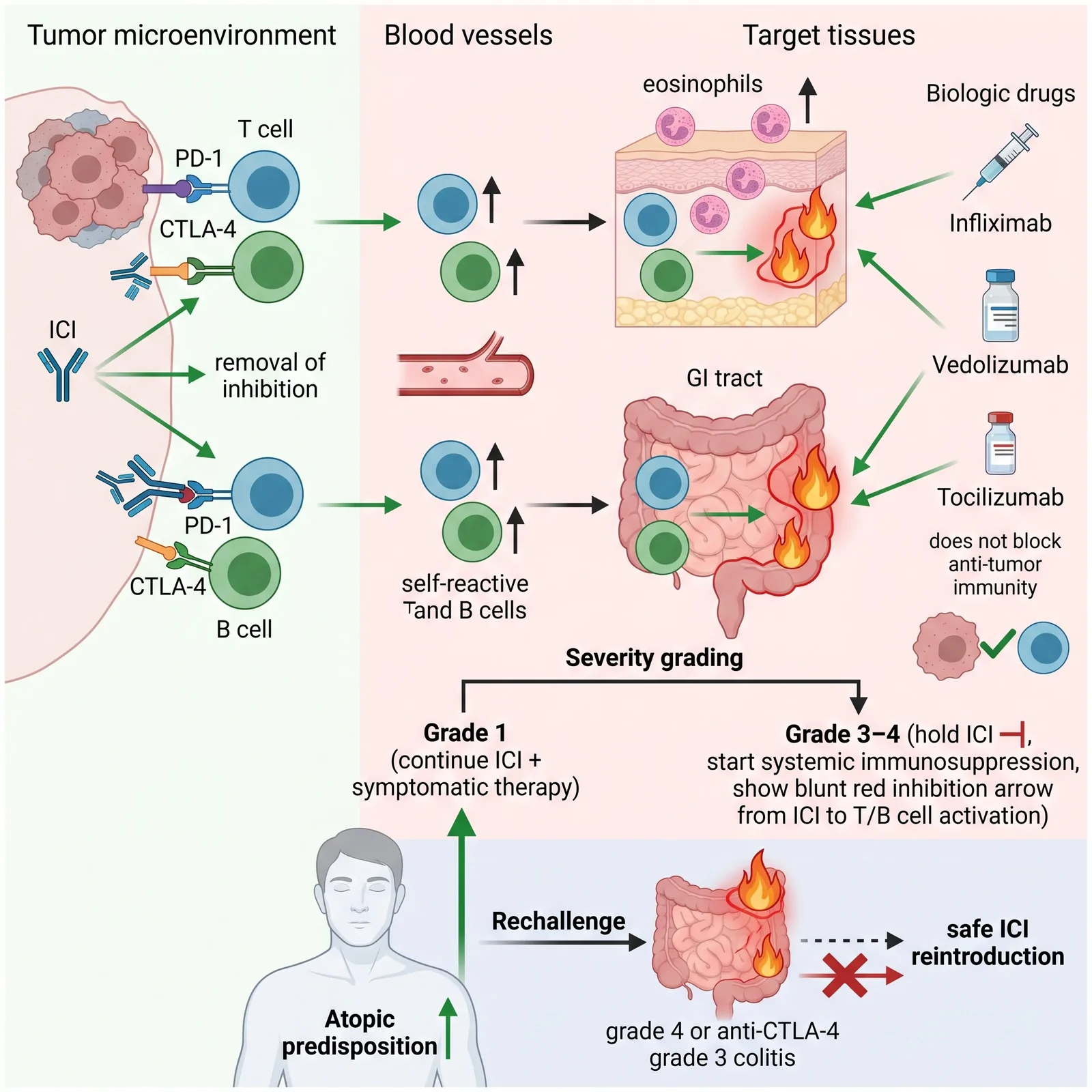
Integrated take-home message: mechanisms and management of ICI-induced cutaneous and gastrointestinal irAEs. The schematic summarizes the central paradigm of ICI-induced irAEs. ICIs remove inhibitory signals from T cells and B cells in the tumor microenvironment, restoring anti-tumor immunity but also unleashing self-reactive T and B cells. These cells migrate to target tissues, including the skin and gastrointestinal tract, where eosinophil infiltration and inflammation occur. Biologic agents for steroid-refractory irAEs include infliximab, vedolizumab, and tocilizumab; tocilizumab is noted not to block anti-tumor immunity. Severity grading guides management: Grade 1 irAEs are managed by continuing ICI with symptomatic therapy, whereas Grade 3–4 irAEs require holding ICI and initiating systemic immunosuppression. Rechallenge is shown in the context of atopic predisposition; grade 4 irAEs or anti-CTLA-4 grade 3 colitis generally preclude safe ICI reintroduction.

## Glossary

ADCC: Antibody-dependent cellular cytotoxicity
ADL: Activities of daily living
ASCO: American society of clinical oncology
BSA: Body surface area
CDC: Complement-dependent cytotoxicity
CMV: Cytomegalovirus
CTCAE: Common terminology criteria for adverse events
CTLA-4: Cytotoxic T-lymphocyte-associated protein 4
DRESS: Drug reaction with eosinophilia and systemic symptoms
ESMO: European Society for Medical Oncology
FMT: Fecal microbiota transplantation
IBD: Inflammatory bowel disease
ICI: Immune checkpoint inhibitor
IFN-γ: Interferon-gamma
IL: Interleukin
IV: Intravenous
NCCN: National comprehensive cancer network
PD-1: Programmed cell death protein 1
PD-L1: Programmed cell death ligand 1
PCR: Polymerase chain reaction
SITC: Society for immunotherapy of cancer
SJS: Stevens-Johnson syndrome
TEN: Toxic epidermal necrolysis
TNF: Tumor necrosis factor.
